# Pterostilbene Fails to Rescue Insulin Secretion and Sensitivity in Multiple Murine Models of Diabetes

**DOI:** 10.3390/nu14183741

**Published:** 2022-09-10

**Authors:** Mads V. Damgaard, Sara L. Jepsen, Stephen P. Ashcroft, Jens J. Holst, Jonas T. Treebak

**Affiliations:** 1 Novo Nordisk Foundation Center for Basic Metabolic Research, Faculty of Health and Medical Sciences, University of Copenhagen, 2200 Copenhagen, Denmark; 2 Department of Biomedical Sciences, Faculty of Health and Medical Sciences, University of Copenhagen, 2200 Copenhagen, Denmark

**Keywords:** diabetes, pterostilbene, streptozotocin, β-cell stress, insulin resistance, pancreas perfusion, insulin secretion, C57BL6/NTac, TALLYHO/JngJ, resveratrol

## Abstract

Diabetes incidence is rising globally at an accelerating rate causing issues at both the individual and societal levels. However, partly inspired by Ayurvedic medicine, a naturally occurring compound called pterostilbene has been demonstrated to protect against diabetes symptoms, though mainly in rats. The purpose of this study was to investigate the putative protective effect of pterostilbene on the two main aspects of diabetes, namely insulin resistance and decreased insulin secretion, in mice. To accomplish this, we employed diet-induced obese as well as streptozotocin-induced diabetic C57BL/6NTac mice for fasting glucose homeostasis assessment, tolerance tests and pancreas perfusions. In addition, we used the polygenic model of diabetes TALLYHO/JngJ to assess for prevention of β-cell burnout. We found that the diet-induced obese C57BL/6NTac mice were insulin resistant, but that pterostilbene had no impact on this or on overall glucose regulation. We further found that the reported protective effect of pterostilbene against streptozotocin-induced diabetes was absent in C57BL/6NTac mice, despite a promising pilot experiment. Lastly, we observed that pterostilbene does not prevent or delay onset of β-cell burnout in TALLYHO/JngJ mice. In conjunction with the literature, our findings suggest variations in the response to pterostilbene between species or between strains of species.

## 1. Introduction

Diabetes is a serious chronic disease arising from insulin insufficiency, either as a result of peripheral insulin resistance, decreased insulin secretion or both. Despite being one of the most researched metabolic diseases, the incidence of diabetes is not only rising but accelerating and is likely to continue this development for the foreseeable future. In 2010, it was estimated that the number of adults with diabetes would reach 439 million globally in 2030 [1]. This estimate was surpassed in 2017 [2]. From a societal perspective, this means an increasing economic burden [3], while for the affected individuals, diabetes can result in various comorbidities such as hypertension, hyperlipidemia and cardiovascular disease [4]. However, diabetes is not a novel problem, so it stands to reason that various people have attempted to remedy this disease. Ayurvedic medicine has for centuries prescribed an aqueous extract of heartwood from the Indian kino tree, *Pterocarpus Marsupium* (PM), to people suffering from glycosuria and wasting. Intriguingly, in recent years such an extract has been reported to be effective in treating diabetic symptoms in rats irrespective of whether the heartwood or bark is used to prepare the PM extract [5,6]. Naturally, this sparked an interest in isolating the active compounds from the PM extract, and pterostilbene (PT), a methylated analogue of resveratrol with increased bioavailability [7], was found to be a promising candidate [8]. This promise has since been confirmed multiple times in streptozotocin (STZ)-induced diabetic rats [9,10,11,12,13]. Mice are not as extensively investigated in this regard, but one study showed similar effects on STZ-induced diabetes in Swiss Albino mice [14]. Moreover, it was reported very recently that PT protects against glycosuria and increased fasting blood glucose as well as kidney dysfunction in high-fat-fed C57BL/6J mice [15]. Thus, PT is an intriguing compound for the treatment of diabetes, and the main aim of the current study was to investigate pterostilbene in relation to the different facets of diabetes, namely insulin resistance and decreased insulin secretion. To do so, we utilized C57BL/6NTac mice in both a diet-induced insulin-resistant setting as well as an STZ-induced insulin-deficient setting. In addition, we investigated whether PT would benefit the TALLYHO/JngJ mouse strain, which is a polygenic diabetes model [16]. The TALLYHO/JngJ model presents with early insulin resistance and a resulting, gradually developing hyperinsulinemia. Over time, the hyperinsulinemia reaches a plateau when β-cell degranulation and loss set in, eventually causing large increases in blood glucose levels in the male mice [17]. This is the initial stage of β-cell burnout, which eventually results in a reversal of the hyperinsulinemia and, if untreated, the wasting and death of the animals. While much remains to be elucidated when it comes to the TALLYHO/JngJ mice and their diabetes variant, the phenotype is reminiscent of human type 2 diabetes, which made it an interesting model for our purposes [18]. Unfortunately, PT did not rescue insulin sensitivity or prevent insulin deficiency in CBL57/6NTac mice and furthermore failed to prevent or delay β-cell burnout in TALLYHO/JngJ mice.

## 2. Materials and Methods

### 2.1. Ethical Approval

All experiments were performed in accordance with European Directive 2010/63/EU of the European Parliament and the Council for the Protection of Animals used for Scientific Purposes. The Danish Animal Experiments Inspectorate gave ethical approvals (#2018-15-0201-01397) and (#2018-15-0201-01558).

### 2.2. Animal Care and Use

All mice were kept single-caged at 20–22 °C and 50% humidity with a 12-h light/dark cycle. They had ad libitum access to food and water. We utilized three different cohorts of male mice for the experiments herein. Both C57BL/6NTac cohorts were acquired from Taconic and the TALLYHO/JngJ cohort from the Jackson Laboratory. The diet-induced obese C57BL/6NTac cohort arrived at 26 weeks of age and received a Western diet with or without 400 mg PT per kg diet (Research diets, D18053102—a D12079B variant using lard and soybean oil as fat sources). They received this diet for a total of 19 weeks (2 weeks of acclimatization and 17 weeks of monitoring) prior to STZ treatment. After STZ treatment, the mice were monitored for an additional six weeks prior to pancreas perfusion. This procedure was terminal. The young C57BL/6NTac cohort arrived at 6 weeks of age and received an Open Standard Diet (Research Diets, D11112201) with or without 800 mg PT per kg diet for 6 weeks prior to STZ treatment. They were terminated by cervical dislocation after the indicated 12 weeks. The TALLYHO/JngJ mice likewise arrived at 6 weeks of age and received the same Open Standard Diet as above with or without PT. Due to animal welfare considerations in relation to the diabetic phenotype, each TALLYHO/JngJ mouse was terminated by cervical dislocation immediately after confirmation that our criteria for β-cell burnout had been reached.

### 2.3. Fasted Glucose Homeostasis Assessment

For assessment of fasting blood glucose and insulin, mice were fasted for two hours, starting two hours into the light phase, and were bled from the tail. Blood glucose was measured using the Contour XT glucometer (Bayer Health Care). For insulin, 5 µL whole blood was deposited directly into a prepared 96-well plate from the Ultra-sensitive Mouse Insulin ELISA Kit (Crystal Chem) using a positive displacement pipette (Microman E, 325 μL, Gilson, Middleton, WI, USA). The analysis was performed in accordance with the manufacturer’s instructions. The measurement of insulin using this approach has been validated previously [19].

### 2.4. Tolerance Tests

Glucose tolerance tests were performed by oral gavage of 2 mg glucose (Fresenius Kabi, Bad Homburg, Germany) per gram of bodyweight after 4 h of fasting, which was initiated two hours into the light phase. Blood glucose levels were measured by tail bleeding 0, 15, 30, 45, 60, 90 and 120 min after gavage using the Contour XT glucometer (Bayer Health Care, Leverkusen, Germany) or the StatStrip Xpress glucometer (Nova Biomedical, Waltham, MA, USA) in cases where blood glucose was out of range of the Contour XT. Additionally, in the young C57BL/6NTac cohort, 5 µL blood samples were taken after 0, 15, 30 and 45 min for assessment of glucose-stimulated insulin secretion. Insulin was analyzed as described above. For the insulin tolerance test, mice were fasted for 4 h with the same onset as above. This was followed by intraperitoneal injection of insulin (Actrapid, Novo Nordisk, Bagsværd, Denmark) at a dosage of 1.5 U/kg. Blood glucose was measured 0, 20, 40, 60, 90 and 120 min after insulin injection.

### 2.5. Streptozotocin Treatment

STZ (Sigma-Aldrich) was dissolved in 20 mM citrate buffer at pH 4.5 and administered subcutaneously twice with a two-day interval to mice that had been fasted in the same way as for the tolerance tests. The initial dosage was 75 mg/kg and the follow up dosage was 50 mg/kg in accordance with a previous study that produced the desired phenotype [20]. In the obese C57BL/6NTac cohort, one mouse from each STZ group was euthanized after STZ treatment for animal welfare reasons (i.e., loss of bodyweight and signs of distress).

### 2.6. Pancreas Perfusion

The perfusion of the pancreas has been described previously [21]. In short, mice were anaesthetized with intraperitoneal injection of Ketamine/Xylazine (0.1 mL/20 g) (Ketamine 90 mg/kg (Ketaminol Vet, MSD Animal Health, Kenilworth, NJ, USA), Xylazine 10 mg/kg (Rompun Vet, Bayer Animal Health). The abdomen of the mouse was cut open and the colon and the small intestine was removed, except for the most proximal part of the intestine (1–2 cm) which shares blood vessels with the pancreas. The spleen and stomach was tied off and removed and the kidneys were ligated but left in the mouse. A catheter (BD Insyte Autoguard, 24 GA 0.75 IN, 0.7 × 19 mm, BD) was inserted into the abdominal part of the aorta allowing for vascular perfusion of the pancreas via the celiac and the superior mesenteric arteries. The perfusion buffer was a modified Krebs-Ringer bicarbonate buffer, which was pH adjusted to ~7.5, heated to 37 °C and gassed with a 95% O_2_/5% CO_2_ mixture during the experiment. The buffer contained 0.1% BSA (Merck KGaA, Darmstadt, Germany), 5% Dextran T-70 (Dextran Products Limited, Toronto, ON, Canada), 3.5 mM glucose and 5 mM each of pyruvate, fumarate and glutamate and the flowrate was 1.5 mL/min. For collection of the effluent, a similar catheter as the one placed in the aorta was inserted into the portal vein. When the catheters were in place, the mice were euthanized by perforation of the diaphragm. After a calibration period of 25 min the experiment was started, and samples were collected every minute using a fraction collector. During the experiment the concentration of glucose was gradually increased every 20 min from 3.5 mM to 6 mM, and lastly 20 mM. A total of 10 mM Arginin was used as a positive control by the end of each experiment. Insulin levels were measured by an in-house radioimmunoassay using an antibody raised against porcine insulin, which cross-reacts strongly with mouse, human and rat insulin (ab 2006-3) [22].

### 2.7. β-cell Burnout Criteria

We defined β-cell burnout in the TALLYHO/Jng cohort as having occurred when two consecutive measurements of insulin averaged below 1 µg/L with two concurrent, consecutive blood glucose measurements averaging above 20 mM. The rationale behind this definition was that a mouse with 20 mM blood glucose should maximize insulin secretion and that if maximal insulin concentration falls below 1 µg/L (estimated normal fasting insulin of healthy mouse) it indicates severely restricted insulin production. The rationale for using the average of two consecutive measurements was to limit the potential for error. This delayed the diagnosis but was merely a parallel displacement for all of the mice, and a few mice, which met the criteria for a single measurement, partly recovered insulin production during the subsequent measurement, thereby validating this approach.

### 2.8. Statistics

Statistical analysis was performed using SAS JMP Pro 15 and Graphpad Prism 9. SAS JMP Pro 15 was used for repeated measurement analyses using the MANOVA personality with Repeated Measures as the response specification. GraphPad Prism 9 was used for simple comparisons between two groups using the *t*-test function, multifactor comparisons using the two-way ANOVA function with Sidak’s multiple comparison test and the comparison between the β-cell survival curves using the Kaplan—Meier survival analysis. Statistically significant effects were indicated; STZ: *p* < 0.05 = §, *p* < 0.01 = §§; *p* < 0.001 = §§§; *p* < 0.0001 = §§§§; PT: *p* < 0.05 = †; *p* < 0.01 = ††; *p* < 0.001 = †††; *p* < 0.0001 = †††† and additional statistically significant effects: *p* < 0.05 = *; *p* < 0.01 = **; *p* < 0.001 = ***; *p* < 0.0001 = ****

## 3. Results

### 3.1. Pterostilbene Does Not Affect Glucose Regulation in Diet-Induced Obese C57BL/6NTac Mice

**Figure 1:**
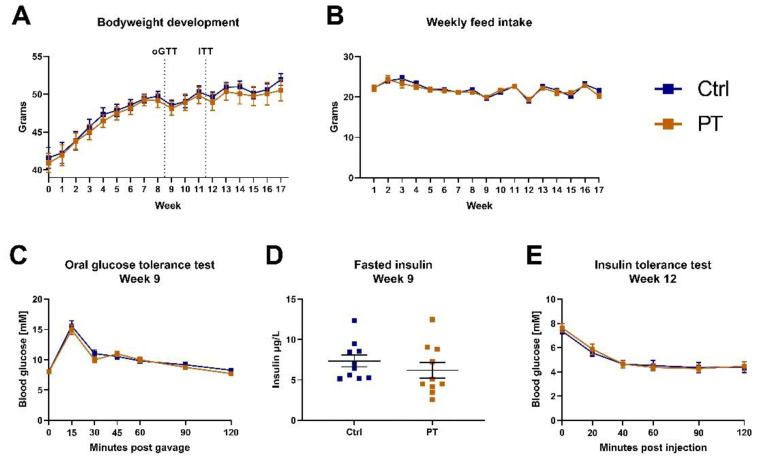
Diet-induced obese C57BL/6NTac cohort (n_ctrl_ = 10, n_pt_ = 10). (**A**) Weekly bodyweight measurements. Western diet was introduced upon arrival, 2 weeks prior to the initiation of the experiment which has been defined as week 0. Timing of oral glucose tolerance test and insulin tolerance test are indicated. (**B**) Total weekly feed intake in grams per mouse (diets were isocaloric). (**C**) Oral glucose tolerance test with 2 mg/kg glucose after a 4-h fast. (**D**) Fasted insulin measurement on blood sampled immediately prior to the oral glucose tolerance test. (**E**) Insulin tolerance test with 1.5 U/kg insulin after a 4-h fast. Data are displayed as mean ± SEM. oGTT, oral glucose tolerance test; ITT, insulin tolerance test; Ctrl, control (group); PT, pterostilbene (group).

Six-month-old, obese, male C57BL/6NTac mice were put on a Western diet upon arrival at our animal facility, and after two weeks of acclimatization, half of the mice were switched to an otherwise identical Western diet containing 400 mg PT pr. kg diet. During the following 17 weeks there were no differences in bodyweight development or feed intake between the two groups (Figure 1A,B). During the 9th week of the experiment, an oral glucose tolerance test was performed, which showed that PT did not affect the glucose tolerance in these mice (Figure 1C). Similarly, the fasting blood insulin was unaffected by PT treatment (Figure 1D), and both groups responded similarly to an insulin challenge (Figure 1E).

### 3.2. A Pilot Streptozotocin Experiment Suggested Protective Effect of PT

**Figure 2:**
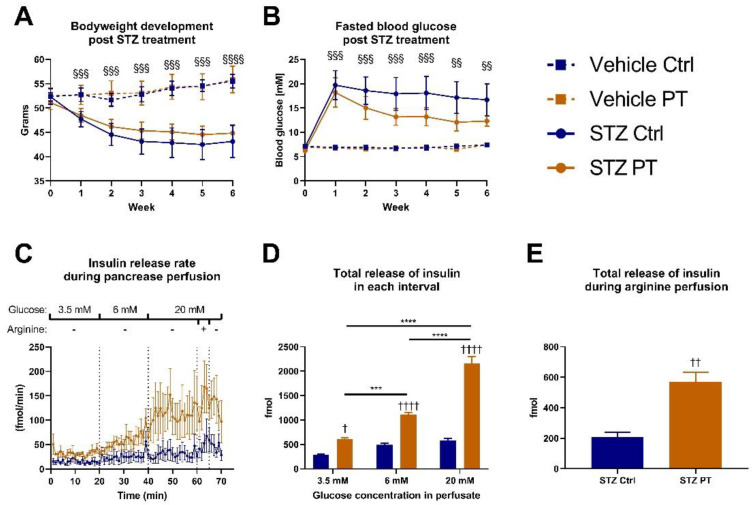
Diet-induced obese C57BL/6NTac cohort (**A**,**B**: n_vehicle ctrl_ = 3, n_vehicle pt_ = 3, n_stz ctrl_ = 6, n_stz pt_ = 6; **C**–**E**: n_stz ctrl_ = 5, n_stz pt_ = 5). Week 0 corresponds to week 17 of the overall experiment. Figure 2**C**–**E** include only STZ-treated mice and one mouse from each group was excluded due to obesity-related complications during surgery. (**A**) Weekly bodyweight measurements after STZ treatment. Week 0 measurement was taken immediately prior to the first dose of STZ. (**B**) Weekly assessment of fasting blood glucose after STZ treatment. Week 0 measurement was taken immediately prior to the first dose of STZ. (**C**) Rate of secretion of insulin in response to indicated concentrations of glucose in the effluent during perfusion of pancreas. Arginine was given at a concentration of 10 mM. (**D**) Total release of insulin during 20 min of exposure to indicated glucose concentrations. (**E**) Total release of insulin during 5 min of exposure to arginine in addition to 20 mM glucose. Statistically significant effects have been indicated; STZ: *p* < 0.001 = §§§; *p* < 0.0001 = §§§§; PT: *p* < 0.05 = †; *p* < 0.01 = ††; *p* < 0.0001 = †††† and additional statistically significant effects: *p* < 0.001 = ***; *p* < 0.0001 = ****. Data are displayed as mean ± SEM. Ctrl, control (group); PT, pterostilbene (group); STZ, streptozotocin; mM, millimolar.

In accordance with the literature, we used the same middle-aged cohort for a pilot experiment in which we administered subcutaneous injections of STZ to a subset of the mice. One week after induction, the mice that received STZ started losing bodyweight compared to the mice that were injected with the vehicle, and the separation between the groups increased weekly (Figure 2A). We observed a slight tendency for the STZ-treated animals to lose less bodyweight if they received PT in the diet, but this did not reach statistical significance. Additionally, the STZ-treated groups separated clearly from the vehicle-treated groups on fasting blood glucose and there was a tendency for the PT treatment to protect against the STZ effect (Figure 2B). Following up, we performed pancreas perfusions on the STZ-treated animals. With this method, we were able to expose the pancreas to varying levels of glucose and measure the resulting insulin output (Figure 2C). By calculating the total release of insulin during each time interval, we observed that the pancreas from the PT-treated mice released more insulin in the hypo-, normo- and hyperglycemic states (Figure 2D). Moreover, as glucose exposure increased, the insulin release followed in a stepwise manner, but this only reached statistical significance in the PT-treated group. Lastly, the pancreas was exposed to arginine as well, to induce maximal insulin release, which again showed that PT afforded the animals some protection against the detrimental effects of STZ on the pancreas (Figure 2E). These promising results encouraged further investigation into the role of PT in protecting against STZ-induced β-cell damage and β-cell stress in general.

### 3.3. Pterostilbene Does Not Protect Young C57BL/6NTac Mice against Streptozotocin-Induced Diabetes

**Figure 3:**
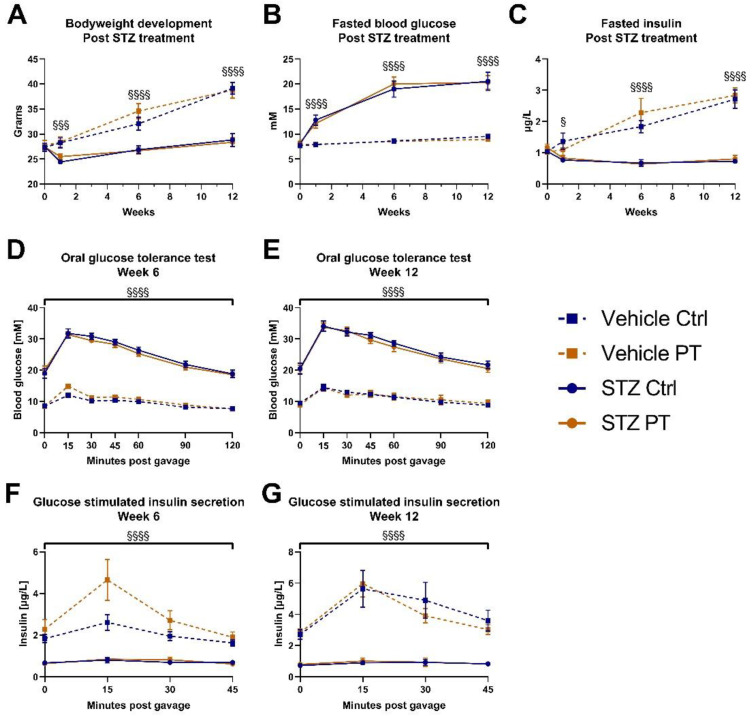
Young C57BL/6NTac cohort (n_vehicle ctrl_ = 9, n_vehicle pt_ = 10, n_stz ctrl_ = 9, n_stz pt_ = 10). Week 0 was immediately prior to first dose of STZ (**A**) Bodyweight measurements at indicated timepoints after STZ treatment. (**B**) Fasted blood glucose at indicated timepoints after STZ treatment. (**C**) Fasted blood insulin at indicated timepoints after STZ treatment. Fasted blood glucose and insulin from weeks 6 and 12 were measured as part of the oral glucose tolerance tests in those weeks. (**D**) Oral glucose tolerance test with 2 mg/kg glucose after a 4-h fast, 6 weeks after STZ treatment. (**E**) Oral glucose tolerance test with 2 mg/kg glucose after a 4-h fast, 12 weeks after STZ treatment. (**F**) Blood insulin in response to glucose gavage during oral glucose tolerance test in week 6. (**G**) Blood insulin in response to glucose gavage during oral glucose tolerance test in week 12. Statistically significant effects have been indicated; STZ: *p* < 0.05 = §; *p* < 0.001 = §§§; *p* < 0.0001 = §§§§. Data are displayed as mean ± SEM. Ctrl, control (group); PT, pterostilbene (group); STZ, streptozotocin.

We followed the pilot STZ experiment up with a similar experiment utilizing a larger sample size. For this purpose, we acquired a cohort of 6-week-old male C57BL/6NTac mice. Half of them received PT in the diet (800 mg/kg) for 6 weeks, before treatment with STZ. The groups had similar average weight prior to STZ treatment, but the mice receiving STZ quickly separated out and the differences increased over the following 12 weeks (Figure 3A). We observed no protective effect of PT in this regard. Similarly, the fasting blood glucose was elevated one week after STZ treatment, and increased further in the following weeks, with no impact of PT (Figure 3B). Fasted insulin levels were decreased by STZ as expected and, irrespective of PT treatment, those that had received STZ were unable to match the increase in insulin secretion of the vehicle groups during the following weeks (Figure 3C). In weeks 6 and 12, we performed oral GTTs with measurement of glucose-stimulated insulin secretion. The results were remarkably similar between the two experiments. In both GTTs there was a clear separation with STZ treatment at all time points, but no effect of PT (Figure 3D,E). For the assessment of glucose-stimulated insulin secretion, the STZ-treated mice were unable to increase insulin output in response to the glucose challenge and had significantly lower insulin output than the other groups at all time points (Figure 3F,G). In week 6, there was a tendency among the vehicle-treated mice for PT to increase insulin secretion, but this did not reach statistical significance and was not replicated in week 12.

### 3.4. Pterostilbene Does Not Protect TALLYHO/Jng Mice against β-Cell Burnout

**Figure 4:**
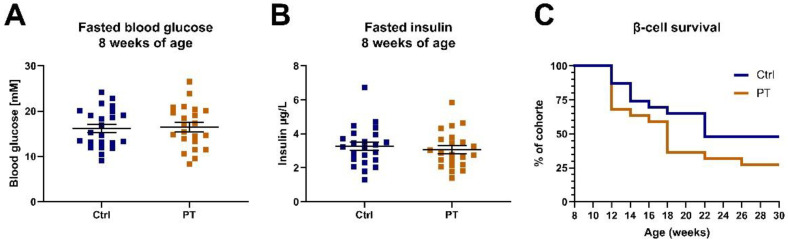
TALLYHO/JngJ cohort (n_ctrl_ = 23, n_pt_ = 22). (**A**) The 2-h fasting blood glucose immediately prior to initiation on the indicated diets. (**B**) The 2-h fasting blood insulin immediately prior to initiation on the indicated diets. (**C**) β-cell survival curve with survival defined as not having reached our criteria for β-cell burnout. Data are displayed as individual data points with an overlay of mean ± SEM. Ctrl, control (group); PT, pterostilbene (group).

Since STZ treatment elicits a somewhat artificial diabetes variant, we decided to expand our investigation by assessing for protection against a more physiological β-cell burnout. We acquired a cohort of TALLYHO/JngJ mice, which, unlike C57BL/6NTac mice, develop β-cell burnout over time. At 8 weeks of age these mice were separated into two groups, balanced for fasting blood glucose and insulin levels, and were given a diet with or without 800 mg PT pr. kg diet (Figure 4A,B). We followed the development of diabetes in these mice by assessing fasting blood glucose and insulin every two weeks for 30 weeks to assess the timing of β-cell burnout. There were no statistically supported differences in the rate at which β-cell burnout occurred between the groups, but, somewhat contrary to expectations, we observed a tendency for PT to be detrimental in this regard (Figure 4C).

## 4. Discussion

With these experiments, we set out to elucidate the putative benefits of PT on diabetes. Initially, we investigated a middle-aged cohort of C57BL/6NTac mice that had been on high-fat diet their entire life, however, the results showed that PT did not improve obesity, glucose tolerance or insulin sensitivity. A previous study corroborated these results, showing that PT did not improve the insulin response, and mildly improved the glucose tolerance only while the mice were young [23]. Likewise, we previously showed that PT failed to protect against developing hyperinsulinemia and additionally had no impact on muscle insulin sensitivity or bodyweight increase [24]. Furthermore, a rat study with a high-fat and high-fructose diet showed that PT was associated with improvements in triglyceride and cholesterol levels as well as a reduction in the markers of liver damage AST and ALT, but PT did not affect bodyweight or serum glucose [25]. In contrast, a recent paper focusing on diabetic kidney disease in high-fat fed C57BL/6J mice showed that in addition to kidney disease, PT protected against obesity, glycosuria and fasting hyperglycemia [15]. Interestingly, in the current study the overall appearance of the glucose excursions during the oral glucose tolerance test suggested that both groups handled the exogenous glucose bolus well due to the rapid return towards normoglycemia. While seeming counterintuitive, it is well established that glucose tolerance in mice can increase with age as a result of increasing systemic insulin levels [26,27,28,29,30]. Reinforcing this, we observed quite high insulin levels in both groups, irrespective of PT treatment. In fact, both middle-aged, obese groups displayed approximately six-fold higher fasting blood insulin than we observed in the young, healthy cohort herein as well as in a comparable previous study [24]. Accordingly, when we performed an insulin tolerance test 3 weeks later, we doubled the regular insulin dose to 1.5 U/kg, which still elicited only a modest insulin response that remained unaffected by PT. Taken together, the results suggested that the middle-aged cohort exhibited a compensated insulin resistance, which was not affected by the addition of PT to the diet.

We elected to use the same middle-aged cohort for a pilot experiment on the interaction between PT and STZ in C57BL/6NTac mice. The results from the pilot looked promising with a tendency for the PT-receiving mice to lose less bodyweight and increase the fasting blood glucose to a lesser degree in response to the STZ treatment. Indeed, when we perfused the pancreas with varying levels of glucose, we observed that the ability to increase insulin secretion in response to glucose was protected in the PT-receiving mice, whereas the other group produced exceedingly small amounts of insulin irrespective of the level of glucose it was exposed to. It should be noted that this pilot experiment was performed with a small sample size (n_vehicle_ = 3, n_stz_ = 5) and the STZ responses are generally heterogenous. Consequently, a follow-up experiment with an increased sample size was warranted. However, when this was conducted, we saw the expected effects of STZ without any impact of PT despite using an increased dose. The discrepancy between the results of the two experiments could conceivably stem from the differences in diet, bodyweight, age or even PT dose, but could also result from an STZ response artefact in the pilot in conjunction with the small sample size. Prior to our study, though often investigated in rats, only two studies were conducted focusing on the putative benefits of PT on STZ-induced diabetes in mice. One study resulted in two publications, but blood glucose was not reported in either of them [31,32]. The other study showed a remarkable rescue of glucose tolerance as well as of fasting blood glucose and insulin [14]. There are, however, multiple differences between that study and ours. Firstly, they used Swiss Albino mice rather than C57BL/6NTac mice and opted for a different STZ-dosing regimen. It is well known that not all mouse strains respond equally to STZ, so the experimental impact of these differences are difficult to gauge [33]. Furthermore, they gave relatively low amounts of PT (~eight-fold lower), but via intraperitoneal injection rather than in the diet. The solution used for this intraperitoneal administration was not disclosed, but could be relevant due to the highly liposoluble status of PT.

As a complement to our STZ experiment, we investigated an additional model of β-cell stress, namely TALLYHO/JngJ mice. This model is interesting due to its similarity to how type 2 diabetes develops in humans. First, a phenotype of insulin resistance is developed, then increasing hyperinsulinemia and associated β-cell stress and eventually β-cell burnout, resulting in rampant blood glucose. We used this model to assess whether PT could delay or prevent the onset of β-cell burnout. To accomplish this, we devised a definition of β-cell burnout based on fasting blood glucose and insulin. Unfortunately, dietary PT failed to delay the onset of β-cell burnout in TALLYHO/JngJ mice, in fact the results were trending in the opposite direction. Importantly, we herein refer to the TALLYHO/JngJ model as a more physiologically relevant variant than STZ-induced diabetes. However, it bears mentioning that it is still a genetic model with the obvious caveat that the important mechanisms for diabetes development may conceivably differ from those of human diabetes.

## 5. Conclusions

We show herein that PT does not protect against insulin resistance in obese, middle-aged C57BL/6NTac mice, and that the protective effect of PT against β-cell stress is absent or circumstantial in this strain of mice. We further show that PT does not protect TALLYHO/JngJ mice from their developing β-cell burnout and ensuing diabetes. Our study in conjunction with the literature on the topic highlights that the previously discovered beneficial effects of PT are likely limited to certain species or strains within species.

## Data Availability

The data presented in this study are openly available in Mendeley Data at DOI: 10.17632/85t6jwgwnz.1.
